# Collagen-derived dipeptide prolyl-hydroxyproline promotes osteogenic differentiation through Foxg1

**DOI:** 10.1186/s11658-017-0060-2

**Published:** 2017-12-01

**Authors:** Yoshifumi Kimira, Haruka Odaira, Kaho Nomura, Yuri Taniuchi, Naoki Inoue, Sachie Nakatani, Jun Shimizu, Masahiro Wada, Hiroshi Mano

**Affiliations:** 1 Faculty of Pharmaceutical Sciences, Josai University, 1-1 Keyakidai, Sakado-shi, Saitama, 350-0295 Japan; 2 Nitta Gelatin Inc., Peptide Division, 2-22 Futamata, Yao, Osaka, 581-0024 Japan

**Keywords:** Prolyl-hydroxyproline, Collagen peptide, Osteoblast differentiation, Foxo1, Foxg1

## Abstract

Prolyl-hydroxyproline (Pro-Hyp) is one of the major constituents of collagen-derived dipeptides. We previously reported that Pro-Hyp promotes the differentiation of osteoblasts by increasing Runx2, osterix and Col1α1 mRNA expression levels. Here, to elucidate the mechanism of Pro-Hyp promotion of osteoblast differentiation, we focus on the involvement of Foxo1 in osteoblast differentiation via Runx2 regulation and the role of Foxg1 in Foxo1 regulation. The addition of Pro-Hyp had no effect on MC3T3-E1 cell proliferation in Foxo1- or Foxg1-knockdown cells. In Foxo1-knockdown cells, the addition of Pro-Hyp increased ALP activity, but in Foxg1-knockdown cells, it had no effect on ALP activity. An enhancing effect of Pro-Hyp on the Runx2 and osterix expression levels was observed in Foxo1-knockdown cells. However, no enhancing effect of Pro-Hyp on osteoblastic gene expression was observed when Foxg1 was knocked down. These results demonstrate that Pro-Hyp promotes osteoblastic MC3T3-E1 cell differentiation and upregulation of osteogenic genes via Foxg1 expression.

## Background

Collagen peptides (CPs) are formed through the hydrolysis of collagen and are widely used as a functional food [1, 2]. Several food-derived collagen oligopeptides were identified in human blood after oral ingestion of CPs [3, 4]. The effects of CPs on bone metabolism were also reported [5–7]. Wu et al. reported that CPs improve bone mineral density in rats fed a calcium-deficient diet [8]. Oral administration of CPs to ovariectomized rats or mice was also shown to increase bone strength and bone mass [9–11]. These reports show that CP plays an important role in bone metabolism.

Prolyl-hydroxyproline (Pro-Hyp) is a major CP component that remains in human blood after the ingestion of CPs [12–14]. Pro-Hyp or hydroxyproline-containing peptides are difficult to hydrolyze in vivo and can play important functions in target tissues [15]. Pro-Hyp reportedly affects the proliferation of fibroblasts and regulates the differentiation of chondrocytes [16, 17].

Regulation of growth factors or transcriptional factors is known to be important for bone repair and cartilage regeneration. We previously reported that Pro-Hyp regulates osteoblast differentiation through Runx2 mRNA upregulation [18]. Runx2 induces osteoblast differentiation and determines the lineage of osteoblasts from multipotent mesenchymal cells, making it a master transcription factor for osteoblast differentiation [19]. Several transcription factors regulate the expression of Runx2 [20]. Forkhead box O1 (Foxo1) belongs to a transcription factor family characterized by a DNA-binding domain called the Fox region, which binds to the Runx2 promoter region and promotes Runx2 transcriptional activity and osteoblast differentiation [21, 22]. FoxG1 is a highly expressed transcriptional repressor in neurons. It negatively regulates the interaction between Foxo1 and Smad, even after activation by extracellular transforming growth factor β (TGF-β) signaling [23, 24].

To reveal more about the mechanism of Pro-Hyp control of osteoblast differentiation, we focus here on the involvement of Foxo1 in osteoblast differentiation via Runx2 regulation and the role of Foxg1 in Foxo1 regulation.

## Methods

### Reagents

Pro-Hyp (Bachem) with a purity of 99% was dissolved in alpha-modified Eagle’s medium (αMEM; Gibco/Life Technologies) and stored at −20 °C. Fetal bovine serum (FBS) was purchased from Sigma-Aldrich. Foxo1 siRNA, Foxg1 siRNA and control siRNA were purchased from Santa Cruz Biotechnology. Anti-Runx2 (cat. no. 8486), anti-Foxo1(cat. no. 2880), β-actin (cat. no. 4970) and secondary antibody (cat. no. 7076) were purchased from Cell Signaling Technology, Inc. Anti-Foxg1(cat. no. ab18259), anti-osterix (cat. no. ab22552), and anti-osteocalcin (cat. no. ab93876) were purchased from Abcam. Anti-Col1α1 (cat. no. sc-8784) was purchased from Santa Cruz Biotechnology.

### Cell culture

MC3T3-E1 cells, a clonal osteoblastic cell line isolated from mouse calvariae, were kindly provided by Dr. Hakeda of the Meikai University School of Dentistry in Sakado, Japan [25]. Cells were cultured in α-MEM containing 10% FBS (Gibco/Life Technologies) and 100 U/ml penicillin. Cell cultures were maintained at 37 °C in a humidified atmosphere of 5% CO_2_ in air.

### Transfection siRNA into MC3T3-E1

MC3T3-E1 cells were plated in 96- or 6-well plates in αMEM containing 10% FBS, transiently transfected with Foxo1, Foxg1 or control siRNA (10 nM) using Lipofectamine Reagent (Life Technologies), and then cultured in the presence or absence of Pro-Hyp (0.1 mM). This study was conducted according to the ethics regulations of Josai University.

### Cell proliferation

Cell proliferation was evaluated using the WST-1 method (Cell Counting Kit; Dojindo Laboratories). Cells were seeded at a density of 3.0 × 10^3^ in each well of a 96-well plate and cultured overnight. Cells were transfected with siRNA and cultured for 2 days in the presence or absence of 0.1 mM Pro-Hyp. After incubation, the absorbance was measured at 450 nm using a microplate reader (Perkin Elmer, Inc.)

### Alkaline phosphatase activity

Cells were seeded at a density of 3.0 × 10^3^ in each well of a 96-well plate and cultured overnight. Cells were transfected with siRNA and cultured for 5 days in the presence or absence of 0.1 mM Pro-Hyp. After incubation, cells were fixed with 20% formalin on ice for 20 min and incubated in 0.05 mol/l 2-amino-2-methyl-1-propanol (AMP) buffer (pH 9.8), containing 10 mM naphthol AS-BI phosphate and 1 mM fast red violet LB salt for 30 min at 37 °C. The staining solution was aspirated and the cells were washed with deionized water [26]. The alkaline phosphatase-stained area was scanned using an image scanner and quantitatively analyzed using ImageJ software.

### Quantitative real-time PCR

MC3T3-E1 cells were seeded in a 6-well plate at a density of 4 × 10^4^ cells/well. After 24 h culture, the cells were transfected with siRNA and cultured for 2 days in the presence or absence of 0.1 mM Pro-Hyp. After incubation, total RNA was extracted from the cells using TRIzol reagent (Invitrogen). First-strand cDNA was converted with the PrimeScript reagent kit (Takara Bio Japan). Quantitative real-time PCR was performed using the TaqMan gene expression assay (Applied Biosystems). The TaqMan probes were: Runx2 (Mm00501584_m1), osterix (Mm04209856_m1), Col1α1 (Mm00801666_g1), Foxo1 (Mm00490671_m1), Foxg1 (Mm02059886_s1), and osteocalcin (Mm03413826_mH). β-actin (Mm02619580_g1) was used as an internal control for normalization of target gene expression.

### Western blot analysis

MC3T3-E1 cells were seeded in a 6-well plate at a density of 4 × 10^4^ cells/well. After 24 h culture, the cells were transfected with siRNA and cultured for 4 days in the presence or absence of 0.1 mM Pro-Hyp. Cells were washed twice with ice-cold PBS and then lysed with RIPA buffer consisting of 25 mM Tris-HCl (pH 7.6), 150 mM NaCl, 1% NP-40, 1% sodium deoxycholate and 0.1% SDS and containing a protease inhibitor cocktail. Cell lysates were centrifuged at 15,000 rpm for 30 min, and the supernatants were collected as the protein samples.

The protein concentration of each sample was measured with BCA Protein Assay Reagent (Thermo Pierce). Proteins were separated via 10% SDS-PAGE and transferred to PVDF membranes. The membranes were blocked with 2% BSA in TBS-T consisting of 10 mM Tris-HCl (pH 7.4), 1.37 M NaCl and 0.1% Tween 20 for 30 min at room temperature. The membranes were probed with antibodies against Foxo1, Foxg1, Runx2, osterix, Col1α1, osteocalcin and β-actin for 1 h at room temperature or overnight at 4 °C. Horseradish peroxidase-conjugated rabbit anti-mouse IgG was applied as the secondary antibody for 1 h at room temperature. Labeled proteins were detected with EZ west Lumi plus (ATTO). Protein bands were analyzed using EZ capture MG (ATTO).

### Statistical analysis

The results are presented as means ± standard deviations (SD). After performing one-way analysis of variance, the Tukey post hoc test was used to compare differences between the means at the 5% probability level (*p* < 0.05).

## Results

### Effects of Pro-Hyp on cell proliferation in Foxo1 knockdown cells

**Fig. 1:**
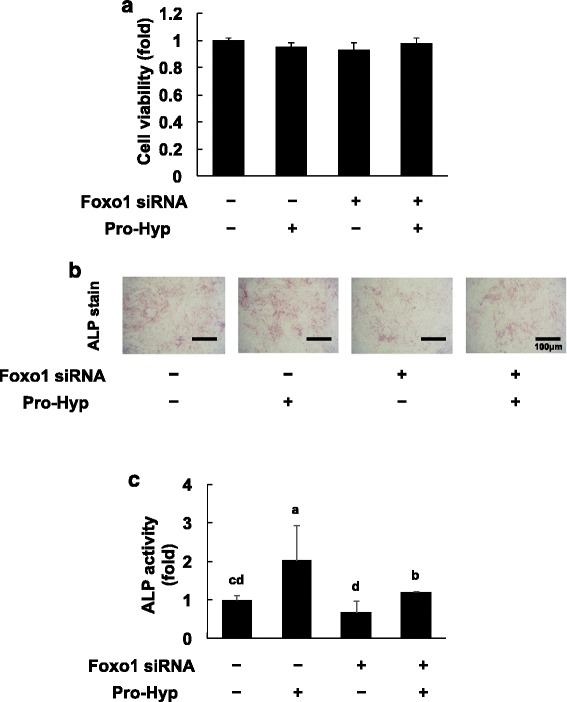
Effect of Pro-Hyp on proliferation and ALP activity in Foxo1-knockdown cells. **a** – Foxo1 was knocked down in MC3T3-E1 cells, which were then cultured with or without 0.1 mM Pro-Hyp for 2 days. MC3T3-E1 cell proliferation was measured using the WST-1 assay. **b** – Foxo1 was knocked down in MC3T3-E1 cells, which were then cultured with or without 0.1 mM Pro-Hyp for 5 days. The scanned images show ALP staining in the cells. **c** – The ALP staining area was evaluated using Image J software. Data are presented as means ± SD (*n* = 4). Bars not sharing a letter differ, *p* < 0.05

We examined the effects of Pro-Hyp on cell proliferation in Foxo1-knockdown cells. Pro-Hyp did not affect MC3T3-E1 cell proliferation and knockdown of Foxo1 did not change the proliferation of MC3T3-E1 cells. Furthermore, cell proliferation did not change with or without Pro-Hyp in Foxo1-knockdown cells (Fig. 1a).

### Effects of Pro-Hyp on ALP activity in Foxo1-knockdown cells

Next, we examined the effect of Pro-Hyp on alkaline phosphatase (ALP) activity to investigate Pro-Hyp influence on osteoblast differentiation in Foxo1-knockdown cells. Pro-Hyp increased ALP activity in control siRNA-transfected cells. ALP activity in Foxo1-knockdown cells was slightly but not significantly lower than in cells transfected with control siRNA. The addition of Pro-Hyp in Foxo1-knockdown cells increased ALP activity (Fig. 1b).

### Effects of Pro-Hyp on gene expression in Foxo1-knockdown cells

We examined whether the effects of Pro-Hyp on osteogenic gene expression were mediated via the expression of Foxo1. PCR results confirmed that knockdown was successful, as the expression of Foxo1 was clearly reduced.

**Fig. 2:**
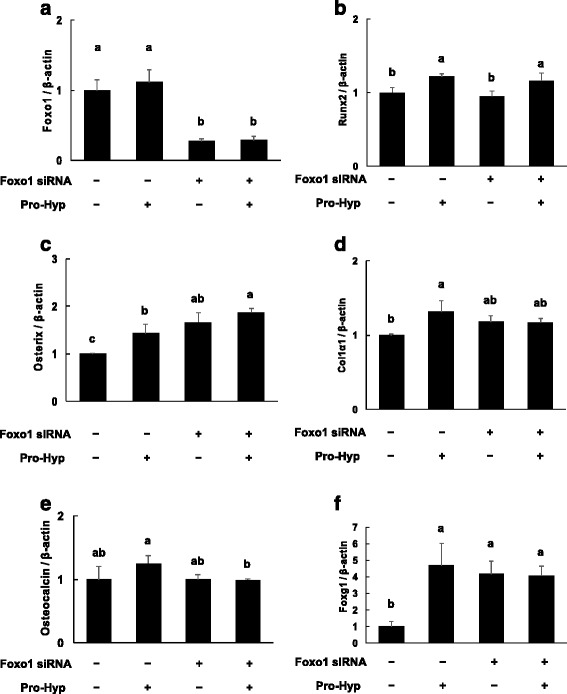
Effect of Pro-Hyp on the mRNA expression levels of Foxo1, Runx2, osterix, Col1α1, osteocalcin and Foxg1 in Foxo1-knockdown cells. Foxo1 was knocked down in MC3T3-E1 cells, which were then cultured with or without 0.1 mM Pro-Hyp for 2 days. After incubation, total RNA was extracted and reverse transcribed, and real-time PCR was carried out. The mRNA expression levels of Foxo1 (**a**), Runx2 (**b**), osterix (**c**), Col1α1 (**d**), osteocalcin (**e**) and Foxg1 (**f**) are shown. Data are presented as means ± SD (*n* = 4). Bars not sharing a letter differ, *p* < 0.05

The addition of Pro-Hyp did not affect Foxo1 mRNA expression level (Fig. 2a). Knockdown of Foxo1 induced expression of osterix mRNA, but not that of Runx2, Col1α1 or osteocalcin mRNA (Fig. 2b–e). In this experiment, knockdown of Foxo1 did not affect the expression of Runx2.

The addition of Pro-Hyp in control siRNA-transfected cells increased the expression level of Runx2, osterix and Col1α1 mRNA, but not that of osteocalcin mRNA. Furthermore, Pro-Hyp increased the expression level of Runx2 mRNA, but not that of osterix, Col1α1 or osteocalcin mRNA in Foxo1-knockdown cells. Foxg1 mRNA expression was significantly increased in Pro-Hyp-treated cells (Fig. 2f). Furthermore, Foxg1 mRNA expression was increased in Foxo1-knockdown cells. Foxg1 mRNA expression was not changed with or without Pro-Hyp in Foxo1-knockdown cells.

### Effects of Pro-Hyp on protein expression in Foxo1-knockdown cells

To examine the effect of Pro-Hyp on the expression of osteogenic proteins in Foxo1-knockdown cells, we used western blotting to evaluate the expression of Runx2, osterix, Col1α1 and osteocalcin as osteogenic proteins. We also examined the influence of Pro-Hyp on the expression of Foxo1 and Foxg1.

**Fig. 3:**
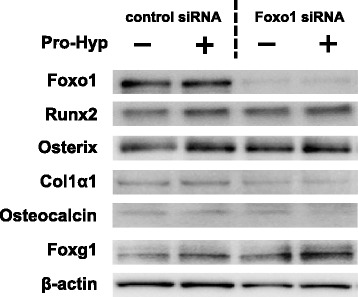
Effect of Pro-Hyp on the protein expression levels of Foxo1, Runx2, osterix, Col1α1, osteocalcin and Foxg1 in Foxo1-knockdown cells. Foxo1 was knocked down in MC3T3-E1 cells, which were then cultured with or without 0.1 mM Pro-Hyp for 4 days. After incubation, cell lysates were collected and expression levels of Foxo1, Runx2, osterix, Col1α1, osteocalcin and Foxg1 were analyzed via western blotting. The data are representative of 3 independent experiments

Western blot results confirmed that knockdown was successful, as the expression of Foxo1 was clearly reduced. The addition of Pro-Hyp did not affect the Foxo1 protein expression level. Knockdown of Foxo1 induced the expression of osterix and Foxg1 protein but did not affect Runx2, Col1α1 or osteocalcin protein levels. The addition of Pro-Hyp in control siRNA-transfected cells increased the expression levels of Runx2, osterix, Col1α1 and Foxg1 protein, but those of not Foxo1 or osteocalcin. Furthermore, Pro-Hyp increased the expression levels of Runx2 and Foxg1 protein, but not those of osterix, Col1α1 or osteocalcin in Foxo1 knockdown cells (Fig. 3).

### Effects of Pro-Hyp on MC3T3-E1 cell proliferation in Foxg1-knockdown cells

Pro-Hyp treatment and knockdown of Foxo1 both increased Foxg1 mRNA expression. Therefore, we examined whether Foxg1 knockdown could change the effects of Pro-Hyp on MC3T3-E1 cell proliferation and differentiation.

**Fig. 4:**
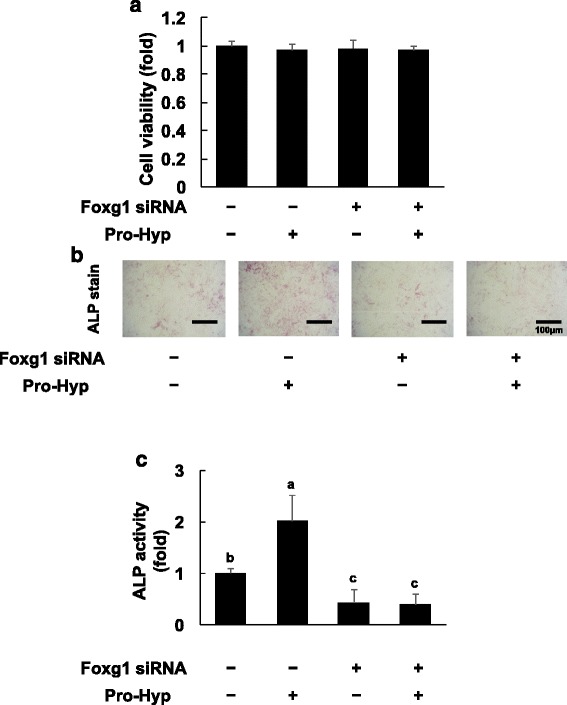
Effect of Pro-Hyp on proliferation and ALP activity in Foxg1-knockdown cells. **a** – Foxg1 was knocked down in MC3T3-E1 cells, which were then cultured with or without 0.1 mM Pro-Hyp for 2 days. MC3T3-E1 cell proliferation was measured using the WST-1 assay. **b** – Foxg1 was knocked down in MC3T3-E1 cells, which were then cultured with or without 0.1 mM Pro-Hyp for 5 days. The scanned images show ALP staining in MC3T3-E1 cells. **c** – The ALP staining area was evaluated using Image J software. Data are presented as means ± SD (*n* = 4). Bars not sharing a letter differ, *p* < 0.05

First, we assessed whether Foxg1 knockdown in MC3T3-E1 cells could change cell proliferation. Knockdown of Foxg1 did not change the proliferation of MC3T3-E1 cells. Furthermore, Pro-Hyp addition did not change cell proliferation in Foxg1-knockdown cells (Fig. 4a).

### Effects of Pro-Hyp on ALP activity under lower levels of Foxg1 in MC3T3-E1 cells

We examined the effect of Pro-Hyp on ALP activity in Foxg1-knockdown cells. ALP activity was significantly inhibited in Foxg1-knockdown cells compared with control siRNA-transfected cells. ALP activity did not change, with or without Pro-Hyp, in Foxg1 knockdown cells (Fig. 4b).

### Effects of Pro-Hyp on gene expression in Foxg1-knockdown cells

**Fig. 5:**
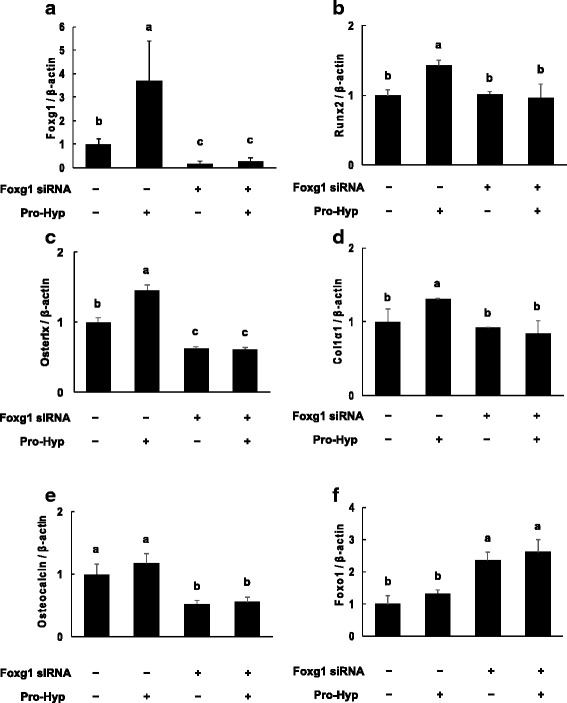
Effect of Pro-Hyp on the mRNA expression levels of Foxg1, Runx2, osterix, Col1α1, osteocalcin and Foxo1 in Foxg1-knockdown cells. Foxg1 was knocked down in MC3T3-E1 cells, which were then cultured with or without 0.1 mM Pro-Hyp for 2 days. After incubation, total RNA was extracted and reverse transcribed, and real-time PCR was carried out. The mRNA expression levels of Foxg1 (**a**), Runx2 (**b**), osterix (**c**), Col1α1 (**d**), osteocalcin (**e**) and Foxo1 (**f**) are shown. Data are presented as means ± SD (*n* = 4). Bars not sharing a letter differ, *p* < 0.05

Foxg1 mRNA was significantly lower in Foxg1 knockdown cells than in control siRNA-transfected cells (Fig. 5a). Foxg1 mRNA expression did not further change, with or without Pro-Hyp treatment, in Foxg1-knockdown cells. Runx2 and Col1α1 mRNA expression did not change in Foxg1-knockdown cells compared with the levels in control siRNA-transfected cells (Fig. 5b and d). Osterix and osteocalcin mRNA expression had significantly decreased in si-Foxg1-transfected cells compared with the levels in control siRNA-transfected cells. Osterix and osteocalcin mRNA expression did not change in the presence or absence of Pro-Hyp in Foxg1-knockdown cells (Fig. 5c and e). Foxo1 mRNA expression was increased in Foxg1-knockdown cells and did not change with or without Pro-Hyp treatment (Fig. 5f).

### Effects of Pro-Hyp on protein expression in Foxg1-knockdown cells

**Fig. 6:**
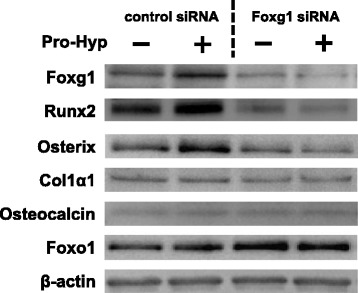
Effect of Pro-Hyp on the protein expression levels of Foxg1, Runx2, osterix, Col1α1, osteocalcin and Foxo1 in Foxg1-knockdown cells. Foxg1 was knocked down in MC3T3-E1 cells, which were then cultured with or without 0.1 mM Pro-Hyp for 4 days. After incubation, cell lysates were collected and the expression levels of Foxg1, Runx2, osterix, Col1α1, osteocalcin and Foxo1 were analyzed via western blotting. The data are representative of 3 independent experiments

We examined the effect of Pro-Hyp on protein expression in Foxg1-knockdown cells. Western blot analysis confirmed successful knockdown, as the expression of Foxg1 clearly decreased. Knockdown of Foxg1 decreased the expression of Runx2 and osterix but did not affect Col1α1 and osteocalcin protein levels. The addition of Pro-Hyp in control siRNA-transfected cells increased the expression levels of Runx2, osterix and Foxg1 protein levels, but not those of Col1α1, osteocalcin and Foxo1. Furthermore, Pro-Hyp did not affect the expression levels of Foxg1, Runx2, osterix, Col1α1, osteocalcin or Foxo1 in Foxg1-knockdown cells (Fig. 6).

## Discussion

In this study, we focused on Foxo1 and Foxg1 proteins and aimed to reveal the osteoblast differentiation control mechanisms of Pro-Hyp. First, we examined the effect of Pro-Hyp on cell proliferation in Foxo1- and Foxg1-knockdown cells. The addition of Pro-Hyp did not change proliferation of those cells, as we found previously [18]. These results indicate that knockdown of Foxo1 and Foxg1 did not change the effect of Pro-Hyp on MC3T3-E1 cell proliferation.

Next, we investigated the effect of Pro-Hyp on osteoblast differentiation in Foxo1- and Foxg1-knockdown cells. There are many reports that Foxo1 regulates the differentiation of osteoblasts [21, 22], but the role of Foxg1 in osteoblasts has not been reported. We used RNAi to suppress gene expression. The expressions of the Foxo1 and Foxg1 mRNA were suppressed on day 2 and those of the protein on day 4, so we collected RNA samples on day 2 and collected protein samples on day 4. Foxo1 knockdown did not affect ALP activity or the expression level of Runx2, the master regulator of osteoblast differentiation, but the expression level of osterix remarkably increased.

In this experiment, it was shown that Foxo1 may not strongly affect osteoblast differentiation. Conversely, knockdown of Foxg1 decreased ALP activity, and Runx2 and osterix expression. These results suggest that Foxg1 plays an important role in osteoblast differentiation.

Furthermore, the Foxg1 expression level increased significantly in Foxo1-knockdown cells and the Foxo1 expression level increased in Foxg1-knockdown cells. Foxg1 binds to the Foxo1–Smad complex, inhibiting its transcriptional activity during neuronal differentiation [24]. We speculate that Foxo1 and Foxg1 regulate each other’s expression, thereby controlling the differentiation of osteoblasts. Since there are no reports of these effects in osteoblasts, this requires further study.

We previously reported that the effect of Pro-Hyp on the ALP activity of MC3T3-E1 cells was well observed 5 days after exposure to Pro-Hyp [18], so we used the same timing for this experiment. Here, the addition of Pro-Hyp increased ALP activity of MC3T3-E1 cells. In Foxo1-knockdown cells, the addition of Pro-Hyp increased ALP activity, but in Foxg1 knockdown cells, this effect was not seen. Pro-Hyp increased Runx2, osterix and Col1α1 mRNA expression, but not that of osteocalcin in control siRNA-transfected cells. Also, Pro-Hyp increased the expression level of Runx2 mRNA in Foxo1-knockdown cells. However, Runx2 and Osterix mRNA expression did not change after the addition of Pro-Hyp to Foxg1-knockdown cells. These results are similar to those from western blotting.

ALP is an early marker enzyme during osteoblast differentiation and is regulated by the expression of Runx2 [27]. Runx2 is a transcription factor that controls skeletal development by regulating the differentiation of osteoblasts and the expression of many extracellular matrix protein genes during osteoblast differentiation [19]. Osterix is also expressed in osteoblasts and is an essential transcription factor in the differentiation of bone marrow mesenchymal stem cells into osteoblasts. Osterix-knockout mice exhibit defects in osteogenesis [28].

Therefore, these data suggest that Pro-Hyp plays a role at an early stage of osteoblast differentiation and Pro-Hyp regulates gene expression of Runx2 and osterix through Foxg1. However, it is unknown whether Foxg1 directly acts on the regulation of osteogenic gene expression by Pro-Hyp. Further study is required.

In summary, this study demonstrated for the first time that Foxg1 has a positive effect on osteoblast differentiation. It was also shown that Pro-Hyp promotes osteoblastic MC3T3-E1 cell differentiation and upregulation of osteogenic genes via Foxg1. However, Foxo1 is not directly related to this action. Our results provide one mechanistic explanation for the osteogenic effects of Pro-Hyp in osteoblasts.

Although further studies are needed to reveal the effect of Pro-Hyp on bone metabolism in vivo, our results suggest that Pro-Hyp or CP consumption could be beneficial in preventing bone-decreasing diseases.

## Abbreviations

ALP: Alkaline phosphatase
AMP: 2-amino-2-methyl-1-propanol
FBS: Fetal bovine serum
Foxg1: Forkhead box G1
Hyp: Hydroxyproline
Pro-Hyp: Prolyl-hydroxyproline Foxo1 Forkhead box O1
Runx2: Runt-related transcription factor 2
αMEM: Alpha-modified Eagle’s medium
